# Dynamic of Circulating DNAM-1+ Monocytes and NK Cells in Patients with STEMI Following Primary Percutaneous Coronary Intervention

**DOI:** 10.3390/jcdd9110395

**Published:** 2022-11-15

**Authors:** Marko Kumric, Hrvoje Urlic, Admira Bilalic, Nikolina Rezic-Muzinic, Angela Mastelic, Anita Markotic, Doris Rusic, Josip A. Borovac, Darko Duplancic, Marina Luetic, Ivan Covic, Tina Ticinovic Kurir, Josko Bozic

**Affiliations:** 1 Department of Pathophysiology, University of Split School of Medicine, 21000 Split, Croatia; 2 Department of Cardiology, University Hospital of Split, 21000 Split, Croatia; 3 Department of Medical Chemistry and Biochemistry, University of Split School of Medicine, 21000 Split, Croatia; 4 Department of Pharmacy, University of Split School of Medicine, 21000 Split, Croatia; 5 Department of Health Studies, University of Split, 21000 Split, Croatia; 6 Department of Internal Medicine, University of Split School of Medicine, 21000 Split, Croatia; 7 Department of Endocrinology, Diabetes and Metabolic Diseases, University Hospital of Split, 21000 Split, Croatia

**Keywords:** myocardial infarction, remodeling, inflammation, DNAM-1, reperfusion, monocyte, natural killer cell

## Abstract

Although the role of inflammation and adverse cardiac remodeling in myocardial infarction (MI) have been extensively explored, gaps in knowledge on the complex interaction between these processes still exist. Data suggest that DNAX accessory molecule-1 (DNAM-1), an activating receptor implicated in NK cell education, may be involved in cardiac remodeling following coronary artery occlusion. In the present study, we aimed to explore the dynamic of DNAM-1+ monocytes and NK cells in peripheral blood in the early phase following reperfusion in patients with ST-elevation MI (STEMI). The study enrolled 49 patients older than 18 years of age diagnosed with STEMI, referred to primary percutaneous coronary intervention (pPCI). Blood samples were obtained at three distinct points (at admission, 3 h, and 24 h after pPCI) and analyzed using flow cytometry. The number of circulating DNAM-1+ monocytes (CD16++ and CD14++) and CD56dimCD16++NK cells was significantly reduced 3 h after pPCI and subsequently returned to initial levels 24 h after procedure (*p* = 0.003, *p* < 0.001, and *p* = 0.002, respectively). Notably, such dynamic was dependent on age of patients. A positive correlation between high sensitivity troponin I levels and number of CD16++DNAM-1+ monocytes in peripheral blood 3 h after pPCI was observed (r = 0.431, *p* = 0.003). In conclusion, in the present study we delineated the post-reperfusion dynamic of DNAM-1-expresing leukocytes. Additionally, we demonstrated that the number of CD16++ DNAM-1+ monocytes correlate with the extent of myocardial injury.

## 1. Introduction

Despite considerable advancements in interventional cardiology, long-term re-percussions succeeding myocardial infarction (MI) still present a major global health burden [1]. In the recent years, the role of inflammation and its close relationship with adverse cardiac remodeling in MI have been extensively explored [2,3]. Specifically, research has focused on inflammatory response following reperfusion, as in contemporary setting, the affected myocardium is usually reperfu sed in less than two hours following ST elevation MI (STEMI) [4]. Nevertheless, there are still some gaps in knowledge as pre-clinical strategies focused on attenuation of detrimental remodeling have so far failed to translate into clinical practice to an adequate degree [5,6]. Monocytes, an essential component linking the innate and adaptive immune system, emerged as important moderators of early post-MI inflammation [7,8,9]. However, monocytes and macrophage maladaptive response has been shown to contribute to adverse cardiac remodeling [10]. Thus, strategies based on modulation of this response have been explored, but yielded disappointing results [6]. Notwithstanding, such efforts showed that previous models describing dynamics of monocytes post-MI were flawed and underlined remarkable heterogeneity and plasticity in macrophage development, phenotype, and function in ischemic milieu [11]. Although traditional views on inflammation in ischemic heart do not endorse the effect of NK cells, recent evidence underscores NK cell importance in the pathophysiology of MI [12,13]. DNAX accessory molecule 1 (DNAM-1), also known as CD226, is a 67-kDa protein belonging to the immunoglobulin superfamily of receptors constitutively expressed on T cells, monocytes and NK cells [14]. DNAM-1 serves as an activating receptor but is also involved in NK cell education [15]. Recent data imply that DNAM-1 may be involved in the healing and therefore remodeling following coronary artery occlusion [16].

Considering the importance of cardiac remodeling and inflammation in the infarcted heart, whilst having in mind heterogeneity of these processes, we aimed to explore the dynamics of DNAM-1+ monocytes and NK cells in peripheral blood in the early phase following reperfusion in patients with STEMI. In addition, we explored if such dynamics are associated with the extent of myocardial injury, myocardial function and its inter-dependence with various clinical data.

## 2. Materials and Methods

### 2.1. Study Design and Ethical Considerations

The present study was performed at the Department of Cardiology, University Hospital of Split, Croatia, during the period from June 2018 to April 2021.

The study was approved by the Ethics Committee of University Hospital of Split (Class: 003-08/18-03/0001; Number: 2181-198-03-04-18-0040) and was conducted in accordance with all ethical principles of the Helsinki Declaration from 2013. Prior to study enrolment, every participant was informed about the procedures, course and goal of this research and each signed an informed written consent.

### 2.2. Subjects and Inclusion/Exclusion Criteria

In the present study, we consecutively enrolled patients older than 18 years of age diagnosed with STEMI referred to primary percutaneous coronary intervention (PCI). All patients included in the study were diagnosed and treated in accordance with European Society of Cardiology (ESC) guidelines for treatment of STEMI [4]. Exclusion criteria were: circulatory shock at admission (defined by the current ESC guidelines), active malignant disease, leukopenia, immunosuppressive therapy in the last 6 months, acquired immunodeficiency syndrome (AIDS) and other forms of immunodeficiency, autoimmune disease, and previous MI.

### 2.3. Clinical and Laboratory Evaluations

Physical examination and relevant items from medical history were directly collected from all patients included in the study in the first 24 h upon admission. For measurement of body weight (kg) and height (cm) measurements, we used a calibrated scale (Seca, Birmingham, UK). Body mass index was calculated by the body weight (kg) being divided by height-squared (m^2^). Waist circumference (cm) was measured while standing at the mid-point between the inferior tip of the ribcage and the superior aspect of the iliac crest, whereas hip circumference (cm) was measured at the point providing maximum circumference over the gluteal region, using a tape measure. Waist-to-hip ratio (WHR) was calculated by dividing the waist by the hip circumference. Blood pressure was measured on both arms using sphygmomanometer using standard protocol.

A transthoracic echocardiography was performed in the first 24 h of admission. Echocardiographic measurements were taken while patients were at rest and in the left lateral decubitus position; LV ejection fraction (LVEF) was assessed by the 2D biplane method, according to the modified Simpson’s rule. All images were acquired using the Vivid 9 ultrasound system (GE Medical Systems, Milwaukee, WI, USA) and analyzed with the Echo PAC workstation (EchoPac PC, version 112; GE Medical Systems, Milwaukee, WI, USA), and echocardiography was performed by experienced cardiologist.

All PCIs were performed in the cardiac catheterization laboratory by an experienced interventional cardiologist with high procedural volumes. Treatment strategy was determined by at least 2 interventional cardiologists on a case-to-case basis with respect to the patient’s preferences. Patients with technically challenging coronary lesions or with multivessel coronary artery disease were presented to the Heart Team involving cardiac surgeons and interventional cardiologists. In most of the cases, radial approach for an invasive coronary angiography was used. In all patients, guideline-recommended peri and postprocedural pharmacological therapy was used.

The in-hospital mortality risk was assessed by the Global Registry of Acute Coronary Events (GRACE) score, a scoring system intended to stratify patients with acute coronary syndrome according to the risk of in-hospital and 6-month to 3-year mortality [17].

Peripheral blood samples were collected in test tubes on admission and were either immediately analyzed with flow cytometer at the Department of Medical Chemistry and Biochemistry, University of Split School of Medicine, or at the Department of Medical Laboratory Diagnostics for all other analyses. Blood samples were analyzed by a specialist in medical biochemistry. High-sensitivity cardiac troponin I (hs-cTnI) was determined by chemiluminescent microparticle immunoassay (CMIA) using ARCHITECT STAT High Sensitive Troponin-I assay (Abbott Laboratories, Chicago, IL, USA) according to the manufacturer’s instructions. Other laboratory parameters were measured according to standard laboratory procedures.

### 2.4. Flow Cytometry

Blood samples needed for flow cytometry were collected from all patients. Blood samples at three distinct time points (at admission, 3 h after reperfusion, 24 h after reperfusion) for flow cytometry were obtained in all patients that survived the first day following STEMI. All samples were analyzed in a single laboratory by an experienced biochemist following the same standard procedure. One hundred microliters of the whole blood were pre-treated with an Fc receptor-blocking reagent (Miltenyi Biotec GmbH, Bergisch Gladbach, Germany) to prevent nonspecific binding and were incubated for 20 min in the dark at 25 °C with 20 µL of anti-human-CD14s phycoerythrin-conjugated antibodies (BD Pharmingen, San Diego, CA, USA), 5 µL of Alexa Flour 647 antibodies reactive to human CD226 (BD Pharmingen, San Diego, CA, USA) in a first test tube and 20 µL of phycoerythrin-conjugated antibodies reactive to human CD16 (BD Pharmingen, San Diego, CA, USA), 5 µL of mouse antibodies reactive to human CD56 conjugated with BB700 (BD Horizon, San Diego, CA), and 5 µL of Alexa Flour 647 antibodies reactive to human CD226 (BD Pharmingen, San Diego, CA, USA) in a second test tube. Following the red blood cell lysis with BD Pharm LyseTM solution (BD Biosciences, San Diego, CA, USA) with results in good light scatter separation of lymphocytes and red blood cells. Cells were analyzed by flow cytometry (BD Accuri C6, BD Biosciences, Erembodegem, Belgium). Unstained cell samples were measured and processed as negative controls to set the appropriate regions. Cell acquisition was stopped at 106 cells.

**Figure 1:**
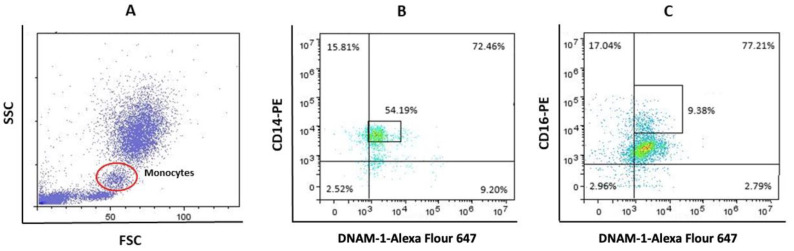
(**A**) Representative gates for monocytes (red ellipse). (**B**) Gating strategy for CD14++DNAM-1+ monocytes. (**C**) Gating strategy for CD16++ DNAM-1+ monocytes.

**Figure 2:**
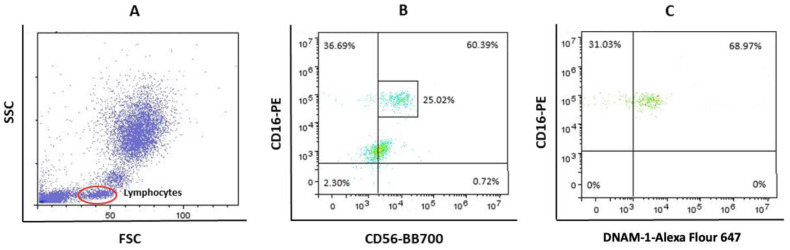
(**A**) Representative gates for lymphocytes (red ellipse). (**B**,**C**) Gating strategy for CD56dimCD16++ DNAM-1+ NK cells.

Data acquired by cytometer were analyzed using the FlowLogic Software (Inivai Technologies, Mentone Victoria, Australia). Leukocyte fluorescence is shown in the forward scatter/side scatter (FSC/SSC) dot plots. FSC parameter indicates cell diameter and SSC indicates cell granularity. Representative dot-plots of gating strategies for CD14++ DNAM-1+ and CD16++ DNAM-1+ monocytes are shown at Figure 1A–C. Monocytes delineated with ellipse E1 (Figure 1A) were analyzed in diagrams CD14 vs. DNAM-1 and CD16 vs. DNAM-1 (Figure 1B, respectively). Within total monocyte population, representative dot-plots are framed 54.19% CD14++CD226+ (Figure 1B) and 9.38% CD16++ DNAM-1+ monocyte subpopulation (Figure 1C). Lymphocytes are delineated with ellipse E1 at Figure 2A. Within total lymphocytes population, in representative CD16 vs. CD56 dot-plot was detected 25.02% CD16++CD56+ NK cells (framed in Figure 2B). Frame from Figure 2B was gate for CD16++CD56+ NK subpopulation which was further analyzed in dot-plot CD16 vs. DNAM-1 with the aim to final detection of percent of DNAM-1 positive CD16++CD56+ NK cells (Figure 2C).

Flow cytometry data were presented either as number of events, i.e., the number of cells detected in the sample, or as Geometric mean fluorescence intensity (GMI) of CD226. GMI was derived by multiplying cell numbers with corresponding fluorescence, adding all results, and dividing the total sum with the total cell number.

### 2.5. Statistical Analysis

The data for this study were analyzed using MedCalc for Windows (version 20.027, MedCalc Software, Ostend, Belgium) and Prism 6 for Windows (version 6.01, GraphPad, La Jolla, CA, USA). Sample size was calculated based on pilot study that included 10 participants. The variable used for this purpose was number of CD14++DNAM-1+ monocytes in peripheral blood sample. With a power of 80% and alpha <0.05, we established that the required number of participants for the present study is 35. Categorical data were represented as absolute numbers (N) and percentages (%), whereas continuous data were shown either as mean ± standard deviation (SD) or median (interquartile range), as appropriate. The normality of distribution was assessed with the Kolmogorov–Smirnov test. To compare whether there was statistically significant difference in number of cells at different measuring points, the Friedman test with post hoc Conover test was employed. To assess association between the extent of myocardial injury and dynamic of leukocyte subsets, we divided patients in two groups depending on the median of hs-cTnI serum levels (99.7 ng/L). Subsequently, we assessed differences in continuous variables between the formed groups using Mann–Whitney U test. Accordingly, we divided patients in two groups depending on the median of age (66 year). Finally, to examine correlation between number of specific subsets of leukocytes in peripheral blood and hs-cTnI levels, we employed Spearman’s rank-order correlation analysis. In this analysis, the r correlation coefficient (rho), two-tailed significance (*p*) values and respective correlation graphs were generated. The *p*-value < 0.05 was considered statistically significant for all analyses.

## 3. Results

In the present study, 49 patients diagnosed with STEMI were included. Characteristics of patients at admission, including age, sex, anthropometric data, vital signs, past medical history, medications, and relevant laboratory findings, were presented in Table 1.

**Figure 3:**
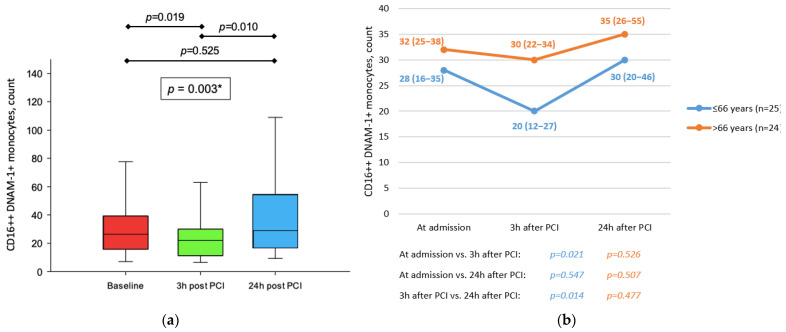
(**a**) Dynamic of CD16++DNAM-1+ monocytes in peripheral blood in patients with STEMI treated with primary PCI. * Friedman test with post hoc Conover test. (**b**) Comparison between dynamic of CD16++CD226+ monocytes in peripheral blood in patients with STEMI treated with primary PCI with respect to age. All data are presented as median (interquartile range).

The total number of CD16++ DNAM-1+ monocytes in peripheral blood was significantly reduced 3 h after reperfusion and subsequently returned to initial values 24 h after procedure (*p* = 0.003) (Figure 3a). Meanwhile, GMI of DNAM-1 on the same subset of monocytes did not change throughout the first day following primary PCI in patients with STEMI (*p* = 0.628). The observed dynamic of peripheral blood CD16++DNAM-1+ monocytes was not present in older patients (>66 y) (*p* = 0.507) nor patients treated with statins (*p* = 0.685) (Figure 3b).

**Figure 4:**
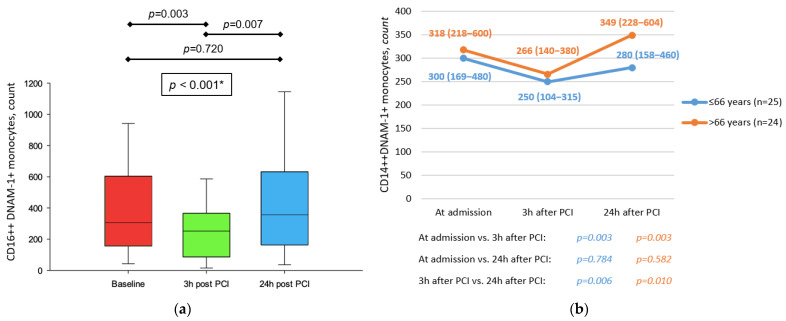
(**a**) Dynamic of CD14++DNAM-1+ monocytes in peripheral blood in patients with STEMI treated with primary PCI. * Friedman test with post hoc Conover test. (**b**) Comparison between dynamic of CD14++CD226+ monocytes in peripheral blood in patients with STEMI treated with primary PCI with respect to age. All data are presented as median (interquartile range).

Number of CD14++DNAM-1+ monocytes in peripheral blood was significantly lower after reperfusion in comparison to baseline, with values returning to that of baseline after 24 h (*p* < 0.001). Conversely, GMI of DNAM-1 on the above-noted subset remained the same after 3 h, but showed an increase after 24 h (*p* < 0.001) (Figure 4a). The above-noted dynamic of peripheral blood CD14++DNAM-1+ monocytes was observed in older patients (>66 y) (*p* < 0.001) but not in patients treated with statins (*p* = 0.271) (Figure 4b).

**Figure 5:**
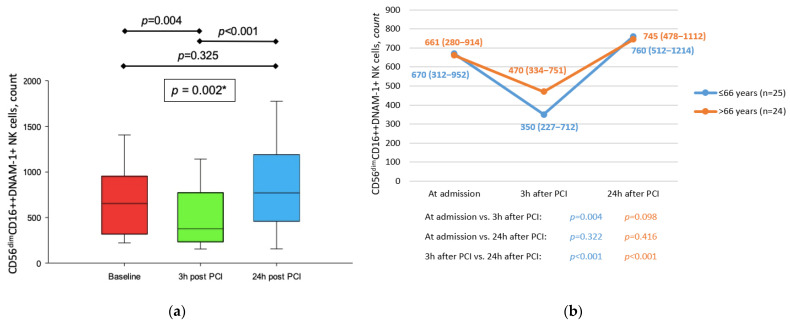
(**a**) Dynamic of CD56dimCD16++DNAM-1+ NK cells in peripheral blood in patients with STEMI treated with primary PCI. * Friedman test with post hoc Conover test. (**b**) Comparison between dynamic of CD56dimCD16++CD226+ NK cells in peripheral blood in patients with STEMI treated with primary PCI with respect to age. All data are presented as median (interquartile range).

Total number of CD56dimCD16++DNAM-1+ NK cells in peripheral blood sample 3 h after reperfusion was reduced in comparison to baseline, with values returning to that of baseline after 24 h (*p* = 0.002) (Figure 5). On the other hand, GMI of DNAM-1 on the above-noted subset of NK cells did not change throughout the first day after MI (*p* = 0.809). Such dynamic of peripheral blood CD56dimCD16++DNAM-1+ NK cells was not observed in older patients (>66 y) (*p* = 0.100) nor patients treated with statins (*p* = 0.527) (Figure 5b).

GMI of DNAM-1 was significantly higher on CD14++ monocytes than on CD16++ monocytes in each measuring point (*p* < 0.001 for all comparisons). Furthermore, GMI of CD226 on NK cells was higher than on both monocyte subgroups in each measuring point (*p* < 0.001 for all comparisons). Summary of dynamics of DNAM-1+ subsets of monocytes and NK cells after STEMI is presented in Table 2.

**Figure 6:**
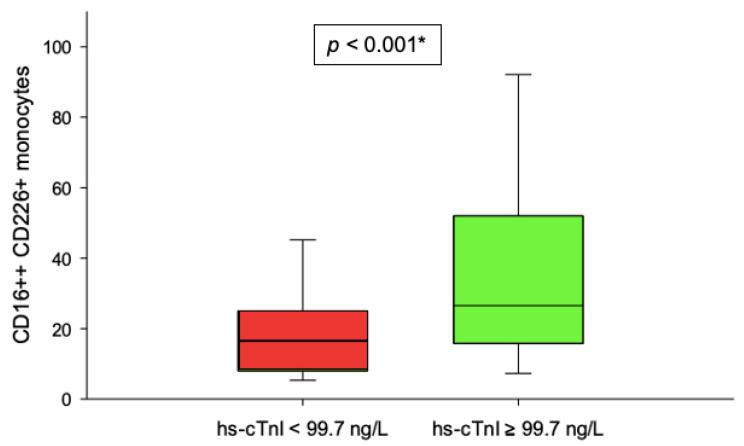
Comparison between number of post-reperfusion CD16++DNAM-1+ monocytes and the extent of myocardial injury. * Mann–Whitney U test.

Patients with lower serum levels of hs-cTnI (<99.7 ng/L) had significantly lower number of CD16++DNAM-1+ monocytes in peripheral blood after reperfusion (3 h) in comparison to patients with higher serum levels of hs-cTnI (≥99.7 ng/L) (*p* < 0.001) (Figure 6), whereas at baseline and at 24 h, no difference existed in number of peripheral blood CD16++DNAM-1+ monocytes (*p* = 0.063 and *p* = 0.144, respectively). Furthermore, there was a moderate positive correlation between serum levels of hs-cTnI and number of CD16++DNAM-1+ monocytes in peripheral blood 3 h after PCI (r = 0.431, *p* = 0.003). On the other hand, number of CD14++DNAM-1+ monocytes and CD56dimCD16++DNAM-1+ NK cells in peripheral blood after reperfusion (3 h) were not associated with hs-cTnI levels (*p* = 0.958 and 0.298, respectively).

The correlation between the risk of in hospital mortality and DNAM-1+ leukocytes was explored using the GRACE score. A moderate negative correlation was established between CD56dimCD16++DNAM-1+ NK cell count and the GRACE score (r = −0.396, *p* = 0.004). On the other hand, neither of the two DNAM-1+ subsets of monocytes significantly correlated with the GRACE score (r = 0.130 and *p* = 0.401 for CD14++, and r = 0.155, *p* = 0.310 for CD16*++* subset). The latter population (DNAM-1+ NK cells), however, showed no correlation with the GRACE score either at admission (r = −0.257, *p* = 0.081) or 3 h after PCI (r = −0.167, *p* = 0.263).

Finally, association between circulating DNAM-1+ leukocytes and LVEF was explored. There was no statistically significant correlation between LVEF and any of the analyzed subsets (Supplementary Table S1).

None of the analyzed monocyte subsets was different between patients who smoke and those who do not at the time of admission: 25.5 (21–36) vs. 27.5 (12–42), *p* = 0.863 for CD16++ DNAM-1+ monocytes; 399.5 (184.5 to 661.5) vs. 278.0 (79.3 to 538.5), *p* = 0.323 for CD14++ DNAM-1+ monocytes and 554.0 (418.8–1009.0) vs. 648.5 (308–874), *p* = 0.791 for CD56dimCD16++DNAM-1+ NK cells. A correlation analysis between C-reactive protein (CRP), a marker of systemic inflammation, and relevant subsets of monocytes and NK cells at baseline demonstrated that DNAM-1+ monocytes but not DNAM-1+ NK cells positively correlate with CRP levels (Supplementary Table S2).

## 4. Discussion

The present study has demonstrated that a peripheral blood count of DNAM-1-expressing monocytes and NK cells reduces after primary PCI in patients with STEMI, with return to initial levels after 24 h. Specifically, such dynamics were observed in CD16++ monocytes, CD14++ monocytes, and CD56dimCD16++ NK cells. To the best of our knowledge, this is the first study to explore the role of DNAM-1+ leukocytes in patients with STEMI.

There is a paucity of data concerning the role CD226 in ischemic myocardium. CD226, or DNAM-1, was initially discovered as an adhesion molecule that is constitutively expressed on T lymphocytes and NK cells, where it plays a role in the T cell-mediated and NK cell-mediated cytotoxicity in tumors [14]. Subsequently, CD226 was also found to promote inflammation, as demonstrated on mice models of dermal fibrosis in systemic sclerosis and acute graft-versus-host disease [18,19]. In addition, recent data suggest that CD226 is implicated in NK cell education [15]. Specifically, Wagner et al. demonstrated that CD226 supports educated NK cells with enhanced effector functions, yet it is not indispensable for its education, as CD226^−/−^ mice are lacking self-responses and NK cells with a normal receptor repertoire. A rather beneficial circumstance for investigating the CD226 in humans is the fact that CD226 is a conserved sequence in human and mouse genes [20]. The role of CD226 in pro-inflammatory monocyte subsets has, so far, been well-documented. Data suggest that this activating receptor is highly expressed on inflammatory monocytes where it is involved in cell adhesion to CD155-expressing cells [21]. Vo et al. showed that CD226 endows transmigration of inflammatory monocytes in the inflamed tissues through endothelial cells in both mice and humans [22]. On the other hand, expression of CD226 on patrolling monocytes in blood is somewhat limited, but it is to be acknowledged that the role of CD226 in these monocytes is very poorly explored [14]. The available data are in line with our findings, as we clearly demonstrated that GMI of CD226 is markedly higher on pro-inflammatory monocytes and NK cells in comparison to nonclassical patrolling monocytes [22].

We argue that the initial drop of the peripheral blood DNAM-1+ CD16++ monocytes following coronary reperfusion is a result of pooling of these monocytes in the infarcted myocardium. A pivotal study that supports these findings was performed by Li et al. [16]. Namely, Li et al. demonstrated on a mice model that CD226 expression is enhanced in the infarcted myocardium. Interestingly, it has been shown that CD226 attenuation tends to alleviate M2 macrophage polarization and suppress M1 type cells, as well as to create a reparative healing microenvironment that subsequently leads to better recovery. Hence, we explored whether the number of DNAM-1+ expressing monocytes and NK cells will depend on cardiac injury. Surprisingly, we demonstrated that number of DNAM-1+ CD16++ monocytes correlates with greater extent of myocardial injury. Namely, post-reperfusion levels of DNAM-1+ CD16++ monocytes positively correlated with hs-cTnI levels. As no correlation was observed with CD14++ monocytes nor CD56dimCD16++ NK cells, both of which have pro-inflammatory phenotype, we may hypothesize that CD226 expressed on non-classical monocytes may exhibit different effects in comparison to pro-inflammatory counterparts [23]. Nevertheless, we found no correlation between number of DNAM-1+ leukocytes and LVEF in our study. Furthermore, correlation analysis demonstrated that CD56^dim^CD16++DNAM-1+ NK cell count 24 h after primary PCI negatively correlates with the GRACE score, a validated indicator of in-hospital mortality risk. Using the same assumptions that reduction in peripheral blood count indicates pooling of cells in the infarcted myocardium, we can speculate that the increase pooling of this pro-inflammatory NK cell subset confers worse outcomes in STEMI patients. Of important note, Jung et al. have employed time-lapse analysis of monocyte flow in the infarcted heart of mouse using miniature suction-assisted endoscope capable of imaging cellular events in the beating heart [8]. The analysis showed that pro-inflammatory monocytes were summoned in the infarcted area but not in remote sites of the heart in the first 3 h following MI, which is contrary to the prevailing conventional belief that the recruitment of monocytes is preceded by that of neutrophils in the setting of MI. Additionally, the authors demonstrated that the origin of early-infiltrating monocytes is mostly peripheral blood, and that monocytes in blood are subsequently replenished with monocytes from the spleen, thus rendering our hypothesis on monocyte recruitment viable.

Although subanalyses from the present study implied that none of the observed dynamics of DNAM-1+ leukocyte subsets are present in patients treated with statins, only 18 included participants were treated with statins, and these results should be interpreted with caution. Nevertheless, in future studies, this observation could be further explored as immunomodulatory effects of statins are well-established and such aspect may be valuable for more comprehensive insight [24]. Specifically, statins can mitigate expression of multiple adhesion molecules (P-selectin, VLA4, ICAM-1) and secretion of proinflammatory cytokines (IL-1B, TNF, IL-6, IL-8), as well as reduce monocyte activation [25,26]. By these mechanisms, statins could thereby change the pattern of inflammatory cell summoning in the infarcted myocardium. On the other hand, as demonstrated by multiple authors, patterns of post-reperfusion inflammatory response seem to be age-related [27,28]. Specifically, it seems that older patients tend to exhibit lower cytokine and chemokine expression in comparison to younger counterparts, and relative contribution of neutrophils in the post-reperfusion patterns of inflammation seems to be replaced by other cell populations. In our study, we demonstrated that the described dynamic was not observed for CD16++DNAM-1+ monocytes and CD56dimCD16++DNAM-1+ NK cells. Finally, in light of available evidence suggesting that diabetes mellitus and hypoglycemic medications may affect inflammatory response in MI, we explored whether dynamics of DNAM-1+ leukocytes will be different in this population of patients [29]. However, no differences were observed for either of the explored subsets.

The present study is burdened by several limiting factors. Firstly, in light of the existing evidence on STEMI treatment, it simply is not possible and would be unethical to form a control group of patients with STEMI who do not undergo primary PCI, at least in areas where this procedure is readily available. Furthermore, increase in number of participants would increase the power of the study. In addition, follow-up after 24 h would also be beneficial for further understanding of events occurring after reperfusion. The present study also lacks analysis of certain markers and populations that might provide more comprehensive insight in the pathophysiology of myocardial infarction, such as CD206 marker and CD56^bright^ NK cell subset. In this regard, direct measurement of cytotoxicity and cytokine production would more adequately delineate the effect of DNAM-1 on NK cell function in the early post-reperfusion setting. Finally, in order to assess the real monocyte and NK cell dynamic in the infarcted myocardium, sampling of the myocardial tissue should be performed. Nevertheless, transcatheter biopsy in infarcted myocardium would be quite challenging to perform, especially considering possible adverse events. Perhaps the best solution to establish the role of DNAM-1 in MI would be to explore this subset in in patients with STEMI undergoing coronary artery bypass grafting, as this procedure allows us to obtain myocardial samples without increasing the risk of complications for patients. The future of clinical applicability of these findings may be the development of more targeted therapies.

## 5. Conclusions

In conclusion, for the first time, we have established dynamic of DNAM-1-expressing monocytes and NK cells in peripheral blood of patients undergoing primary PCI after STEMI. In addition, we demonstrated that the number of DNAM-1+ CD16++ monocytes correlate with the extent of myocardial injury, thus highlighting putative implications of this molecule in management of patients with MI. Finally, the obtained data suggest that age may affect the inflammatory response associated with DNAM-1 expressing leukocytes in STEMI. Nevertheless, further research in which DNAM-1 expression in infarcted tissue will be explored is warranted to establish conclusive findings.

## Figures and Tables

**Table 1:** Baseline characteristics of patients.

Variable	STEMI (*n* = 49)	Age > 66 y (*n* = 24)	Age ≤ 66 y (*n* = 25)	*p*
Age, years	66.2 ± 10.9	75.2 ± 6.3	57.6 ± 6.6	<0.001 *
Male sex	35 (71.4%)	16 (62.5%)	19 (76%)	0.474 †
Body mass index, kg/m^2^	27.3 (25.4–29.4)	27.6 (25.3–29.5)	26.9 (25.4–29.0)	0.542 ‡
Waist-to-hip ratio	1.04 (0.98–1.08)	1.02 (0.93–1.07)	1.05 (1.01–1.09)	0.147 ‡
Systolic blood pressure at admission, mmHg	130 (120–147)	138 (125–141)	130 (120–150)	0.818 ‡
Diastolic blood pressure at admission, mmHg	80 (70–86)	80 (68–81)	80 (70–90)	0.095 ‡
Heart rate at admission, bpm	76 (63–88)	70 (60–84)	75 (70–96)	0.045 ‡
Symptom-to-door time, minutes	72 ± 14	73 ± 16	71 ± 15	0.876 *
Door-to-balloon time, minutes	35 ± 9	34 ± 10	36 ± 11	0.563 *
Diabetes mellitus	8 (16.3%)	4 (16.7%)	4 (16%)	1.000 §
Arterial hypertension	24 (49%)			
Smoking	32 (65.3%)	12 (50%)	20 (80%)	0.029 †
Dyslipidemia	7 (14.3%)	3 (12.5%)	4 (16%)	1.000 §
Atrial fibrillation	2 (4.1%)	0 (0%)	2 (8%)	0.489 §
Family history of cardiovascular disease	6 (12.2%)	4 (16.7%)	2 (8%)	0.417 §
History of PCI or CABG	6 (12.2%)	3 (12.5%)	3 (12.0%)	1.000 §
History of CV-related hospitalizations	6 (12.2%)	3 (12.5%)	3 (12.0%)	1.000 §
GRACE score, points	116 (98–140)	131 (118–147)	99 (93–117)	<0.001 ‡
Left ventricular ejection fraction, %	53.5 (47.0–58.5)	53.0 (49.3–58.8)	54.0 (44.5–56.3)	0.733 ‡
Multi-vessel disease	2 (4.1%)	1 (4.2%)	1 (4.0%)	1.000 §
Beta-blocker use	14 (28.6%)	7 (29.2%)	7 (28.0%)	0.929 †
ACE inhibitor or ARB use	22 (44.9%)	9 (37.5%)	13 (52.0%)	0.313 †
Calcium channel blocker use	4 (8.2%)	2 (8.3%)	2 (8.0%)	1.000 §
Statin use	18 (36.7%)	10 (41.7%)	8 (32%)	0.561 §
Diuretic use	10 (20.4%)	6 (25%)	4 (16%)	0.496 §
Acetylsalicylic acid use	4 (8.2%)	2 (8.3%)	2 (8.0%)	1.000 §
C-reactive protein, mg/L	4.1 (2.0–11.6)	3.4 (2.0–11.8)	5.6 (2.0–11.8)	0.624 ‡
hs-cTnI at admission, ng/L	99.7 (27.4–602.6)	126.2 (33.0–859.7)	68.7 (23.4–513.2)	0.317 ‡
Urea, mmol/L	6.6 ± 1.2	6.9 ± 2.2	6.3 ± 2.1	0.314 *
Creatinine, μmol/L	80 (75–100)	82 (76–103)	78 (73.2–95.8)	0. 378 ‡

Numerical data are presented as mean ± SD or median (IQR) as appropriate. * Student’s *t*-test. † Chi squared test. ‡ Categorical data are presented as *n* (%). § Fisher’s exact test Abbreviations: hs-cTnI, High-sensitivity cardiac troponin I; PCI, percutaneous coronary intervention; CABG, coronary artery bypass grafting; ARB, angiotensin receptor blocker.

**Table 2:** Dynamics of DNAM-1+ subset of monocytes and NK cells after primary PCI in patients with STEMI.

Cell Population	At Admission	After Primary PCI (3 h)	After 24 h	p *
CD16++DNAM-1+ monocytes, *total count*	26 (15.8–38.3)	**19 (8.3–30)**	29 (18.5–54.5)	0.003
CD16++DNAM-1+ monocytes, *GMI of CD226*	2107.75 (1871.89–2464.57)	1952.71 (1751.11–2302.57)	2012,510 (1896.75–2401.35)	0.628
CD14++DNAM-1+ monocytes, *total count*	307 (159.3–595)	**253 (110.8–405.3)**	356 (168.0–615.5)	<0.001
CD14++DNAM-1+ monocytes, *GMI of CD226*	5101.61 (4201.75–5990.25)	4584.74 (3917.05–5413.19)	**5268.19 (4558.42–6055.79)**	<0.001
CD56dimCD16++DNAM-1+ NK cells, *total count*	646 (319–937)	**361 (231.5–769.8)**	764 (458.5–1122.3)	0.002
CD56dimCD16++DNAM-1+ NK cells, *GMI of CD226*	6663.35 (6188.85–7696.27)	7059.55 (5846.46–8865.80)	6738.57 (6173.21–7658.45)	0.809

The data are presented as median (IQR). Bolded values represent values that differ. Abbreviations: GMI, Geometric mean intensity; PCI, percutaneous coronary intervention; NK, natural killer. * Friedman test with post hoc Conover test.

## Data Availability

Study data can be provided upon reasonable request.

## Supplementary Materials

The following supporting information can be downloaded at: https://www.mdpi.com/article/10.3390/jcdd9110395/s1, Table S1: Correlation between left ventricular ejection fraction values and DNAM-1+ subset of monocytes and NK cells in different time points following the primary PCI in patients with STEMI; Table S2: Correlation between C-reactive protein and DNAM-1+ subset of monocytes and NK cells at admission in patients with STEMI.
