# Supplementary material for: Dynamic of Circulating DNAM-1+ Monocytes and NK Cells in Patients with STEMI Following Primary Percutaneous Coronary Intervention
Source: J Cardiovasc Dev Dis. 2022 Nov 15;9(11):395. doi: 10.3390/jcdd9110395 (PMC9693248; doi:10.3390/jcdd9110395)
Supplement: Supplementary file 1 [file jcdd-09-00395-s001.zip › jcdd-1975499-supplementary.pdf]

Supplementary Table S1. Correlation between left ventricular ejection fraction values and DNAM-1+ subset of monocytes and NK cells in different time points following the primary PCI in patients with STEMI.

	Cell Population	r—Correlation Coefficient	*p* *
**At admission**	CD16++DNAM-1+ monocytes, *total count*	0.018	0.907
	CD14++DNAM-1+ monocytes, *total count*	0.282	0.057
	CD56dimCD16++DNAM-1+ NK cells, *total count*	−0.100	0.509
**3h after primary PCI**	CD16++DNAM-1+ monocytes, *total count*	0.163	0.280
	CD14++DNAM-1+ monocytes, *total count*	0.261	0.080
	CD56dimCD16++DNAM-1+ NK cells, *total count*	0.146	0.334
**24 h after primary PCI**	CD16++DNAM-1+ monocytes, *total count*	−0.028	0.019
	CD14++DNAM-1+ monocytes, *total count*	0.114	0.469
	CD56dimCD16++DNAM-1+ NK cells, *total count*	0.116	0.459

Abbreviations: GMI: Geometric mean intensity; PCI: percutaneous coronary intervention; NK: natural killer. * Spearman rank correlation analysis.

Supplementary Table S2. Correlation between C-reactive protein and DNAM-1+ subset of monocytes and NK cells at admission in patients with STEMI.

Cell Population	r—Correlation Coefficient	*p* *
CD16++DNAM-1+ monocytes, *total count*	0.339	0.021
CD14++DNAM-1+ monocytes, *total count*	0.370	0.010
CD56dimCD16++DNAM-1+ NK cells, *total count*	−0.144	0.334

Abbreviations: PCI: percutaneous coronary intervention; NK: natural killer. * Spearman rank correlation analysis.
